# Endometriosis in a Patient with Mayer-Rokitansky-Küster-Hauser Syndrome

**DOI:** 10.1155/2014/376231

**Published:** 2014-12-30

**Authors:** Júlia Kefalás Troncon, Ana Carolina Tagliatti Zani, Andrea Duarte Damasceno Vieira, Omero Benedicto Poli-Neto, Antônio Alberto Nogueira, Júlio César Rosa-e-Silva

**Affiliations:** Ribeirão Preto School of Medicine, University of São Paulo, Avenida Bandeirantes, 3900 Ribeirão Preto, SP, Brazil

## Abstract

*Objective*. To report a case of Mayer-Rokitansky-Küster-Hauser syndrome (MRKH) in which there were two nonfunctional rudimentary uteruses with the presence of ovarian endometrioma, corroborating that there are valid alternative theories to the existence of endometriosis, rather than Sampson's theory alone, such as the coelomic metaplasia theory. *Design*. A case report. *Setting*. A tertiary referral center, which is also a university hospital. *Patient*. A fifteen-year-old patient with MRKH syndrome and endometriosis. *Intervention*. Laparoscopic approach for diagnostic confirmation and treatment of the endometrioma. *Results*. Evidence of endometriosis in a patient with no functional uterus. *Conclusions*. This case report and a few others that are available in the literature reinforce the possibility that coelomic metaplasia could be the origin of endometriosis. Patients with müllerian agenesis and pelvic pain should be carefully evaluated, and the presence of pelvic endometriosis should not be excluded.

## 1. Introduction

Mayer-Rokitansky-Küster-Hauser (MRKH) syndrome is characterized by different degrees of müllerian duct abnormalities, usually with the congenital absence of the upper two-thirds of the vagina, together with uterine agenesis or the presence of a rudimentary uterus. The rudimentary uterus can have a functioning or nonfunctioning endometrium. It is the second most common cause of primary amenorrhea, with a reported incidence of 1 in 4000 female live births [1]. Pelvic pain might be associated with hematometrium in case of a functioning uterus. Distortion of anatomy that accompanies müllerian abnormalities predisposes the appearance of endometriosis, and therefore such patients, when presenting pelvic pain, should always be considered for diagnosis.

Endometriosis is defined as the presence of endometrium-like tissue outside of the uterus, which leads to an inflammatory process. Symptoms include cyclic and acyclic pelvic pain, dysmenorrhea, dyspareunia, and infertility, besides gastrointestinal and urinary complaints that may be present when there is deep infiltrating endometriosis. Diagnosis should be predominantly based on the characteristics of the pain, combined with imaging methods such as ultrasound and magnetic resonance imaging, although the gold standard is laparoscopic visualization and confirmation through biopsy. Treatment is based on medical therapy, which includes both hormonal and nonhormonal drugs such as analgesics, and surgery is also a very effective approach towards pain [2, 3].

There are a few theories that try to explain the mechanism of endometriosis, a condition that is nowadays estimated to affect 10 to 15% of women in reproductive age [4]. One of the most accepted theories is the theory proposed by Sampson, in which endometrial implants occur because of retrograde menstruation. Other theories suggest that there may be endometriotic cells originated from other tissues rather than the endometrium and uterus [5], for example, through coelomic metaplasia, a hypothesis proposed by Meyer that suggests that the original coelomic membrane undergoes metaplasia, forming typical endometrial-like glands and stroma. The histologic findings of gradual transition from normal-appearing ovarian surface epithelium and ovary epithelial inclusions to minimal formation of endometrioid glandular epithelia, as well as the transition from normal ovarian stroma to endometriotic stroma, provide direct evidence supporting coelomic metaplasia in the genesis of ovarian endometriosis [6].

However, endometriosis is a complex disease, and most likely a group of distinct factors combined have an important role in its pathophysiology, such as familial aggregation and genetic polymorphisms, and hormonal interference through estrogen and progesterone receptors [7]. Each of these mechanisms might have a simultaneous part in the etiology of the disease, rather than being individually responsible for endometriosis in different patients. That is, immune and endocrine factors, also called endocrine disrupting chemicals, could promote the differentiation of stem cells and cells of the peritoneum into endometriotic cells [5].

A few authors have reported endometriosis in patients with MRKH. Cho et al. reported a case of endometrioma in a patient with MRKH with no uterus [8]. Mok-Lin et al. also reported endometriosis in a patient with complete uterus agenesis [9]. Some other studies also describe endometriosis in patients with MRKH, but they report a rudimentary uterus that might have a functioning endometrium. Such is the case in the study by Parkar and Kamau, in which there were two horns that did not communicate with the vagina and there was also evidence of functioning endometrium, with adenomyosis, resulting in small hematometra bilaterally [10].

In this case report, we present endometriosis in a patient with Mayer-Rokitansky-Küster-Hauser syndrome, without a functioning uterus. This case reinforces the theory of coelomic metaplasia as having a complementary role in the genesis of endometriosis rather than Sampson's retrograde menstruation theory alone.

## 2. Case Report

A fifteen-year-old patient was seen in 2001 with primary amenorrhea and normal secondary sexual characteristics. Thelarche and pubarche occurred at 11 years of age. Laboratory tests included follicle-stimulating hormone (FSH), luteinizing hormone (LH), dehydroepiandrosterone sulfate (DHEA-S), prolactin (PRL), and thyroid-stimulating hormone (TSH), and they were all within the normal ranges.

Magnetic resonance imaging showed a nonexistent uterus and presence of ovaries bilaterally. In 2002, the patient was submitted to a neovaginoplasty and, in 2003, to a surgical correction of stenosis. She also started using a vaginal mold. The patient remained asymptomatic until 2009 when, with 24 years of age, she appeared with a complaint of abdominal pain. The physical exam was normal and she was then submitted to a pelvic sonography in the beginning of 2010. The exam showed an absent uterus and topic ovaries, with the right one measuring 9,3 cm^3^ and the left one 12,2 cm^3^. In the retrovesical topography, in continuation with the left ovary, there was a hiperecogenic image with debris, capsulated, with little vascularization in the Doppler study, which measured 45 mm.

**Figure 1:**
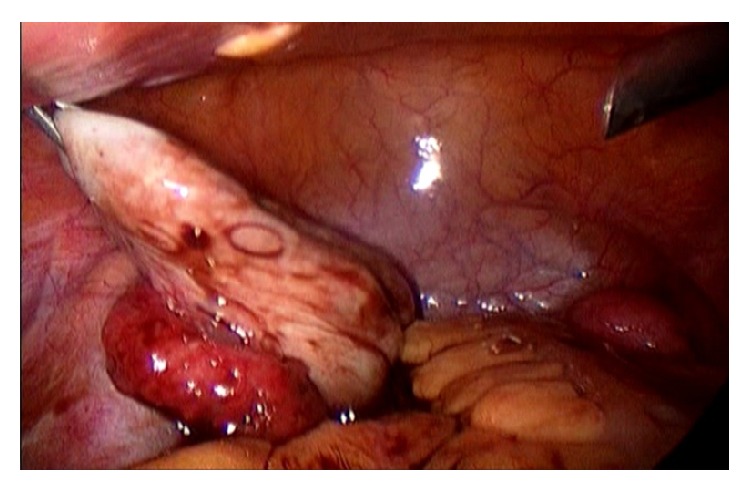
Endometrioma of the left ovary.

**Figure 2:**
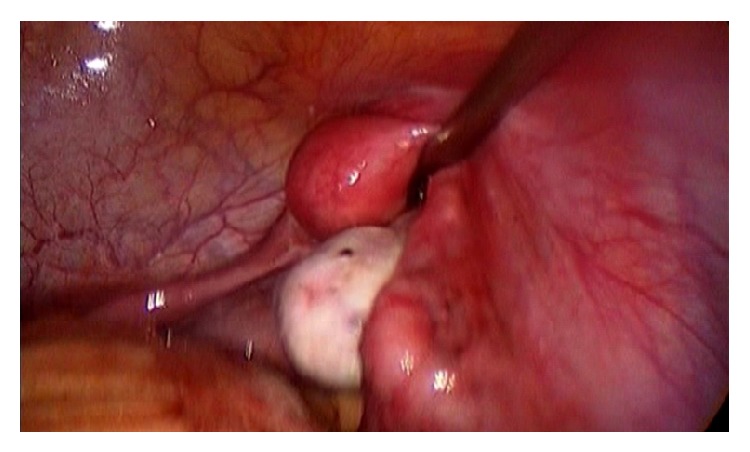
Rudimentary uterus on the right.

In a laparoscopy done in March 2010, two rudimentary uteruses and an endometrioma in the left ovary were observed (Figures 1, and 2). The cyst's capsule was resected and the biopsy confirmed cystic endometriosis. The continuous use of an oral contraceptive was established and the patient became asymptomatic.

In 2012, the patient returned with gestational wish. She underwent ovarian stimulation, followed by successful oocyte retrieval, in vitro fertilization, and embryo transferral to a surrogate mother. Pregnancy was achieved in the second embryo transferral, and, in November 2013, the surrogate mother gave birth to a healthy newborn.

## 3. Discussion

Endometriosis is defined as the presence of tissue similar to the endometrium outside the uterine cavity, which induces a chronic inflammatory reaction, leading to adhesion formation and interference with normal reproductive processes [11]. Multiple hypotheses have been suggested to explain the pathogenesis of endometriosis, including theories of retrograde menstruation [12], lymphatic and vascular metastases [9], immunologic deficiency resulting in insufficient clearance of ectopic endometrial cells [13], and coelomic metaplasia [14]. None of them alone supply a sufficient explanation that can be applied to all cases. This pathology remains an enigma despite the extensive clinical investigations and experience. It is suggested that an endometrioma is a pseudocyst formed by accumulation of menstrual debris from endometrial implants adherent to the peritoneal layer of the ovary, which generate from adhesion of active superficial implants in that peritoneum and posterior invagination [15, 16].

According to the Brazilian Society of Endometriosis and Minimally Invasive Gynecology (SBE), this disease has become, in recent decades, a public health problem, with significant morbidity and unquestionably high costs. According to data from SBE from 2009, currently in Brazil about six million women have the disease. For unknown reasons, both the incidence and aggressiveness of the disease have been increasing alarmingly. However, advances of diagnostic methods and laparoscopic techniques may be one of the factors involved in increased incidence [17].

The prevalence of endometriosis in patients with Rokitansky-Küster-Hauser syndrome (MRKH) without functioning endometrial tissue appears to be very low. A diagnosis of MRKH involves physical examination and imaging modalities. Magnetic resonance imaging (MRI) is the gold standard for the uterus and surrounding structures [18], allowing for better visualization of müllerian structures and better delineation of endometrium than ultrasound. As with our patient, secondary sexual characteristics and external genitalia are generally normal, as are hormonal levels; a vaginal dimple or short, blind-ending vagina and a hymenal fringe are usually present [19]. In the present case, after imaging methods and laparoscopic visualization and approach, diagnosis was obtained, as well as clinical improvement and a satisfactory outcome for the patient, especially with the use of continuous oral contraception.

According to the literature, it is possible to notice that patients with MRKH syndrome who present acute pelvic pain, endometriotic ovarian cysts, or adenomyotic müllerian remnant should be considered for diagnosis; and MRI and laparoscopy are the recommended diagnostic tools and in regard to laparoscopy generally also the treatment.

For a safe laparoscopic approach in women with müllerian abnormalities, according to Will et al., some recommendations should be followed; for example, an adequate preoperative assessment of the urinary tract is imperative, given the high incidence of associated anomalies. Considering the approach of the uterine remnants, medial traction has to be part of the surgical technique to avoid lateral pelvic wall injuries, and the surgeon should be aware of possible vascular anomalous supply [20].

## 4. Conclusions

There are many theories that try to explain the origin of endometriosis. The most accepted is Sampson's retrograde menstruation theory. This theory is reinforced by some studies that report that obstructive müllerian anomalies are more associated with endometriosis, by which an increase in retrograde menstrual flow occurs secondary to obstruction. Sampson's theory is also supported by the observation of the anatomical distribution of endometriotic lesions, which is asymmetric in a way that would be expected after retrograde menstrual flow. This theory cannot explain, however, the appearance of endometriosis in patients with nonfunctioning uteruses, which is the case in some patients with Mayer-Rokitansky-Küster-Hauser syndrome. Even the lymphatic and vascular dissemination theory and the immunologic deficiency theory can be discarded in these cases.

This case report and a few others that exist in the literature reinforce the possibility that coelomic metaplasia could be the origin of endometriosis, described as the transformation of pluripotential cells in endometrial cells in the peritoneal cavity. Patients with müllerian agenesis and pelvic pain should be carefully evaluated, and the presence of pelvic endometriosis should not be excluded.
